# Meeting at the DNA: Specifying Cytokinin Responses through Transcription Factor Complex Formation

**DOI:** 10.3390/plants10071458

**Published:** 2021-07-16

**Authors:** Jan Erik Leuendorf, Thomas Schmülling

**Affiliations:** Dahlem Centre of Plant Sciences, Institute of Biology/Applied Genetics, Freie Universität Berlin, Albrecht-Thaer-Weg 6, D-14195 Berlin, Germany; j.e.leuendorf@fu-berlin.de

**Keywords:** *Arabidopsis thaliana*, cytokinin, type-B response regulator, phytohormone, two-component system, crosstalk, transcription, transcription factor complex

## Abstract

Cytokinin is a plant hormone regulating numerous biological processes. Its diverse functions are realized through the expression control of specific target genes. The transcription of the immediate early cytokinin target genes is regulated by type-B response regulator proteins (RRBs), which are transcription factors (TFs) of the Myb family. RRB activity is controlled by phosphorylation and protein degradation. Here, we focus on another step of regulation, the interaction of RRBs among each other or with other TFs to form active or repressive TF complexes. Several examples in *Arabidopsis thaliana* illustrate that RRBs form homodimers or complexes with other TFs to specify the cytokinin response. This increases the variability of the output response and provides opportunities of crosstalk between the cytokinin signaling pathway and other cellular signaling pathways. We propose that a targeted approach is required to uncover the full extent and impact of RRB interaction with other TFs.

## 1. Introduction

The plant hormone cytokinin has numerous biological activities in regulating plant development and biotic and abiotic stress responses [1,2,3]. Amongst others, cytokinin regulates shoot meristem size and activity [4], leaf size through control of cell division and termination of leaf growth [5,6], leaf senescence [7,8], shoot branching [9], flowering time [10], sex determination [11], root meristem size [12,13] and lateral root formation [14], the response to the availability of various nutrients [15,16], the response to highlight and photoperiod stress [17,18], and the defense against certain pathogens [19,20]. The large variety and versatility of different functions that cytokinin fulfills raises the question of how a single phytohormone can regulate so many different aspects of plant life. At least part of the answer lies in the complex and specific transcriptional responses that are a primary result of cytokinin signaling [21,22,23,24,25,26].

Cytokinin signals through a plant two-component signal transduction system (TCS). This consists of membrane-bound HISTIDINE KINASES (HK), which sense the cytokinin free bases [27] and transmit the signal through a multistep phosphorylation cascade via HISTIDINE PHOSPHOTRANSFER (HP) proteins to nuclear-localized type-B RESPONSE REGULATOR (RRB) proteins, which are the primary CK output TFs. *Arabidopsis thaliana* encodes three cytokinin receptors (AHK2, AHK3 and CRE1/AHK4), five AHPs and eleven ARABIDOPSIS RRB (B-type ARR) proteins. Transcriptomic studies have shown that some of the RRBs convey a very large part of the transcriptional response to CK, which is the basis to realize at the end the various biological functions of the hormone. In this review, we will therefore first summarize current knowledge about RRBs and their CK-dependent gene regulatory activity. However, despite genome-wide coverage of gene expression data it is largely unknown how the context-dependent specificity of the transcriptional response to CK is achieved. Comparison of transcriptomic data, the occurrence of RRB DNA-binding sites and target genes identified by chromatin immunoprecipitation (ChIP) suggested that RRBs may often not alone be responsible for the transcriptional CK output. In its second part, this review reports on the increasing evidence that RRBs may act as dimers or team up with other TFs belonging to different TF families. Several examples from Arabidopsis illustrate that TF complex formation contributes to modulate the CK signaling output and provides opportunities for crosstalk with other pathways.

## 2. B-Type Response Regulators

**Figure 1:**
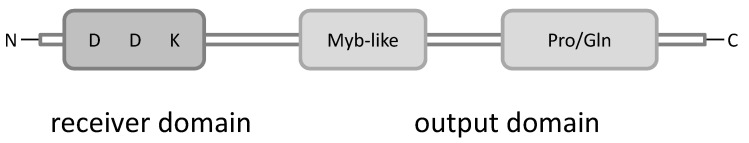
Schematic structure of plant RRB proteins. The N-terminal signal receiver domain contains a DDK motif as the phosphoacceptor site. The C-terminal extension (output domain) contains a Myb-related GARP DNA binding domain (B-motif) and the proline-/glutamine-rich transactivation domain. In addition, RRBs contain at least one nuclear localization signal.

The structure of plant RRBs resembles that of prokaryotic RRs. They have at the N-terminal end a phosphate-accepting receiver domain with a conserved Asp residue as the site for regulatory phosphorylation. There is at least one nuclear localization signal and towards the C-terminal end is located a Myb-like DNA-binding domain followed by a putative Gln-/Pro-rich activation domain (Figure 1) [28,29]. Several RRBs of Arabidopsis have been shown to bind to a short DNA sequence containing the core cytokinin response motif (CRM) A/GGATC [28,30,31]. The best characterized extension motif (ECRM) bound by ARR1 is AAGAT(T/C)TT [32] but variants of this exist [33,34,35].

Based on the evolutionary relationship of their DNA-binding domains the Arabidopsis RRBs have been subdivided into three subfamilies, subfamily B-I (ARR1, ARR2, ARR10, ARR11, ARR12, ARR14 and ARR18), B-II (ARR13, ARR21) and B-III (ARR19, ARR20) [36,37]. A core set of subfamily B-I RRBs, ARR1, ARR10 and ARR12, regulate redundantly a number of important aspects of cytokinin functions, like regulation of growth and development during the vegetative phase [38,39] and responses to high light and photoperiod stress [1,18,40]. Consistently, the transcriptional response to cytokinin was strongly reduced in the *arr1 arr10 arr12* mutant, but it was not completely abolished and the expression of some cytokinin response genes was hardly affected [38]. ARR2 has specific functions during leaf senescence [8,41] and contributes to *Pseudomonas syringae* resistance [19,42]. While there is convincing evidence for these four RRBs of Arabidopsis that they mediate cytokinin activity there is less evidence for several other RRBs.

Overexpression of a truncated version of ARR11 caused phenotypic changes reminiscent of increased cytokinin activity [31] and was able to induce the expression of the *WUSCHEL* (*WUS*) gene, comparable to ARR1, ARR2, ARR10 and ARR12 [43], but a comprehensive loss-of-function study is missing so far. Notably, ARR11 was, unlike ARR1, ARR2, and ARR10, not able to bind to the CRM sequence [31]. Under non-stress conditions, ARR11 together with ARR1 and ARR12 physically interacts with SnRK2 and represses its kinase activity, which shuts down the drought response pathway [44]. Mutation of *ARR14* caused a reduced induction of a cytokinin response gene similar to the consequences of *ARR1* mutation suggesting a role in cytokinin signaling [45]. ARR18 activated a cytokinin reporter gene in a transient assay and its overexpression caused reduced root elongation indicating cytokinin activity [46]. However, neither loss of function of *ARR11*, *ARR18* nor a combination of both caused an altered cytokinin sensitivity of root elongation [47]. Furthermore, neither *ARR11* nor *ARR14* or *ARR18* restored cytokinin sensitivity in the *arr1 arr12* mutant when driven from the *ARR1* promoter, raising the possibility that they might function in a different context [48]. Indeed, ARR11 is involved in the crosstalk of SA and JA affecting the resistance against the pathogen *Botrytis cinerea* [49] and ARR18 acts as a positive regulator of the response to osmotic stress in Arabidopsis seeds [50]. *ARR19* and *ARR20* are predominantly expressed in the chalazal endosperm [51,52] but their functional roles are unknown; their combined mutation did not alter cytokinin sensitivity in root growth or hypocotyl elongation assays [48]. However, ARR19 as well as the distantly related ARR21 were both able to activate the expression of the cytokinin-responsive *ARR6* promoter [53] and their activity pattern on truncated versions of the reporter driving reporter gene expression resembled ARR10 activity [45,53]. Furthermore, *ARR21* expressed under the *ARR1* promoter restored the cytokinin responsiveness of the *arr1 arr12* mutant [48], both being consistent with a function in cytokinin signaling. Taken together, the RRBs of Arabidopsis are functionally different and roles of several RRBs in cytokinin signaling remains to be determined, in particular of those of subfamilies B-II and B-III.

RRBs have also cytokinin-related functions in other plant species, like the regulation of various developmental processes in rice [54,55,56], regulation of nitrogen starvation in wheat [57] and of adventitious root formation in poplar [58].

The activity of RRB proteins is primarily regulated by cytokinin-dependent phosphorylation of the conserved Asp residue in the receiver domain. Phosphorylation relieves the inhibitory effect of this domain and allows RRBs to bind to their target sequences and initiate transcription [31,33]. A further established factor regulating RRB activity is the ubiquitin/26S proteasome protein degradation pathway. For example, the stability of ARR1 and ARR2 is controlled by cytokinin [59,60]. A small family of F-box proteins, the KISS ME DEADLY (KMD) protein family, interact with RRBs to target them for proteasomal degradation [61]. In this way, KMD proteins act as negative regulators of the cytokinin response. In this review we will give an overview on yet another possibility to regulate the transcriptional output of the cytokinin signal, namely RRB dimerization or complex formation with other TFs to diversify the cytokinin output.

## 3. Cytokinin-Regulated Genes

Several studies of the transcriptomic response to treatment with cytokinin revealed a complex response involving the induction and repression of a large number of different genes in Arabidopsis [21,22,23,24,25,26] and in rice [62,63]. Despite variations in the experimental set ups of the different studies, e.g., differences in hormone concentration, age of the analyzed plants or probed plant tissue, a core set of cytokinin-regulated genes could be identified [21,22,24,25]. Among the first induced cytokinin target genes are *RRAs*, which encode negative regulators of the cytokinin transduction system [64,65]. Additional rapidly regulated genes code for cytokinin metabolic enzymes, e.g., CYTOKININ OXIDASE/DEHYDROGENASE (CKX) and CYTOKININ HYDROXYLASE (CYP735A2), indicating that homeostatic control is an important role of the immediate early transcriptional response [21,22,24]. Other immediate early cytokinin response genes belong to different TF families and suggest that downstream cascades of TFs are operating (summarized in [66]). Notably, relevant targets of RRBs expressed in only small domains and meanwhile known to be direct RRB targets like the *WUS* gene [67,68] were not discovered by these global approaches using seedling material. Furthermore, analysis of the root transcriptome or of specific stages of leaf development resulted in the identification of additional cytokinin-responsive genes distinct from those previously reported suggesting that the coverage of cytokinin-regulated genes is as yet not complete [5,25].

Part of the cytokinin-regulated genes identified by transcriptomic analyses were confirmed as RRB in vivo binding sites using ChIP assays [35,68] (see for a detailed analysis [69]). Further, ATAC-seq-based analysis demonstrated that the accessibility of genomic regions near several cytokinin-regulated genes changes in response to cytokinin and that part of these genes overlapped with those identified by RRB ChIP analysis [70]. However, there are a number of aspects of the transcriptional cytokinin response, which cannot be readily explained by RRBs being the only TFs mediating the early transcriptional response to cytokinin.

- Analysis of the occurrence of the cytokinin response motives in cytokinin-regulated genes revealed that the core cytokinin response motif was not overrepresented in cytokinin-responsive promoters [24]. The ECRM AAGAT(C/T)TT [32] was around twofold overrepresented, but nevertheless missing in 55% of the promoters of cytokinin inducible genes. The lack of these only known cytokinin response elements and RRB binding sites in the promoters of these genes raises the question how these genes are regulated by cytokinin [24].
- Of the 226 genes of a core-set of cytokinin-regulated genes, the “Golden list” [22], only 93 were bound by ARR10 in a ChIP assay [68,69].
- Another study identifying ARR1, ARR10 or ARR12 target genes using ChIP assays was unable to show binding of any of these RRBs to 74 of the 226 genes (32.7%) of the “Golden list” [35].
- The same study showed that only a small portion of genes bound by ARR1, ARR10 or ARR12 are transcriptionally regulated by cytokinin (490 out of 3373, i.e., 14.5%) [35]; see also [69].

Taken together, a significant number of cytokinin-regulated genes lack a discernible CRM and a critical number of demonstrably cytokinin-regulated genes are not directly bound and possibly not directly regulated by RRBs. This conclusion can be drawn even when considering that not all RRB binding sites are known and taking into account that ChIP assays will not detect all genes binding RRBs. It is possible that in these instances additional factors, including other TFs, are needed for the regulatory activity of RRBs and might alter their DNA binding properties. Notably, based on the analysis of cis-regulatory elements and promoter activation studies in *rrb* mutants, which showed that several different RRBs were required for full reporter gene activation. Ramireddy et al. [45] proposed a model where the dimerization of RRBs with other RRBs or with other TFs is necessary for full transcriptional activation of cytokinin target genes such as ARR16. Recently, Polko et al. [63] suggested as an explanation for differences in the motifs of up- and down-regulated genes identified in a meta-analysis of the transcriptional responses of rice roots to CK that distinct partner TFs may be involved in mediating gene induction versus gene repression by RRBs.

## 4. Formation of Transcription Factor Complexes: A Way to Modulate TF Activity

The formation of TF complexes is a common regulatory mechanism in prokaryotic and eukaryotic organisms to control gene expression by combining different input information [71,72]. TF complexes are formed either DNA-dependent or -independent by homomeric or heteromeric interaction of TF proteins. The composition of the complex can affect the orientation of TFs to their DNA binding motif and strengthen or weaken the binding properties of the whole complex. In this way the composition of the TF complex can change the accession to different DNA binding motifs and as a result influences the accessibility of target gene promoters which directly affects their transcriptional activity [71,73].

In addition, interactors of TFs may have activating (cooperative) or repressing (antagonistic) characteristics and may increase or decrease DNA occupancy by TFs [71,73,74]. Some TFs require the formation of dimers to become transcriptionally active. In plants, this is often found for members of the BASIC REGION/LEUCINE ZIPPER (bZIP) TF family. BZIP TFs interact via the leucine zipper domain located in their C-terminal α-helices and bind the DNA via the N-terminal basic region, forming a y-shaped TF-TF-complex. While many bZIPs can form homodimers (e.g., members of the bZIP group A, G, H), other bZIPs only or preferentially form heterodimers (bZIP34, bZIP61) to fulfill specific functions [75,76,77]. Here, the change from the monomeric to the oligomeric form and the composition of the dimer is an additional step of regulation of the transactivation properties [71,78].

There are also well-documented examples for cooperating activity of TFs from other plant TF families. The MADS TFs bind generally as dimers to DNA but some form tetramers and bind to two distinct sites, including members of the SEPALLATA clade. The different heteromeric composition of SEPALLATA TF tetramers determines their accession to cis-regulatory elements and in this way controls the expression of different target gene sets. These in turn regulate distinct flower organogenesis programs and are thus the basis for the ABCE model of flower organ formation [79,80,81].

The organization and maintenance of the shoot apical meristem is controlled by the CLAVATA (CLV)-WUS signaling system [82]. The homeodomain TF WUS is responsible for the regulation of *CLV3* gene expression and exists in a monomeric and in a dimeric form. The availability of tandemly arranged binding motifs (e.g., G-box) in the recognition site and a higher WUS protein abundance promotes the formation of the homodimeric state and enhances the binding affinity to the target gene promoter [83,84,85]. Additionally, WUS can form heterodimers with members of the HAIRY MERISTEM (HAM) TF family or with SHOOT MERISTEMLESS (STM) [86,87]. A model described by Gruel and coworkers [88] predicts that in HAM expressing cell layers, WUS forms WUS-HAM heterodimers, which, in contrast to the WUS monomer, represses the expression of CLV3. In contrast, the interaction of WUS and STM can strengthen the binding of WUS to the *CLV3* promoter [86]. The formation of different WUS complexes is one part of the regulatory processes to modulate expression of *CLV3* in order to pattern the SAM and to define the stem cell region [88]. Interaction between distinct HAM TFs and WUS-related proteins may operate also within other plant stem cell niches [87].

Another example of interacting plant TFs is the heterodimerization of KNOTTED-like (KNOX) and BEL1-like (BLH or BELL) homeodomain proteins. KNOX proteins can have different BELL partners, leading to multiple combinations with distinct activities regulating many aspects of plant morphogenesis, including gynoecium development [89]. Additionally, WRKY transcription factors can act also in various homo- and heterodimer combinations to activate or repress gene expression and thereby regulate diverse biological processes [90]. Relative concentrations of interacting TFs can modulate their differential activities as has been shown recently in a model for the interacting TFs SHORTROOT (SHR) and SCARECROW (SCR) and their activities in distinct cell types of the root stem cell niche [91].

Taken together, it is clear that TF complex formation is an important aspect of transcriptional regulation which should also be considered for RRBs. In many prokaryotic two-component systems RRs dimerize with their receiver domain which is thought to change the DNA-binding properties and the transcriptional activity [92,93]. One aspect is the stronger affinity of the dimer/oligomer-complex to the target promoter/DNA. Often the phosphorylation of the RRs changes the structure of the receiver domain, thereby inducing its dimerization [94,95]. In the following sections we are going to describe several cases of plant RRB complex formation illustrating how this contributes to their functional versatility.

## 5. RRBs Dimerize and Interact with Other TFs

The first indication for a possible role of TF complex formation in the cytokinin response came from yeast two-hybrid (Y2H) interaction studies. In the first systematic tests for Arabidopsis RRB interaction, ARR14 formed homodimers and was able to establish heterodimers with ARR2 [96]. Later, additional targeted tests showed that ARR10 and ARR12 can interact with CYTOKININ RESPONSE FACTOR (CRF) proteins (ARR10-CRF6; ARR12-CRF1/2) [97]. In an untargeted Y2H interaction screen, ARR1 was shown to interact with the ETHYLENE RESPONSE FACTOR8 (ERF8) [96,98]. Several large-scale analyses of the Arabidopsis and rice interactomes have identified additional TFs interacting with RRBs. A study using Cre-reporter-mediated yeast two-hybrid coupled with next-generation sequencing (CrY2H-seq) found that at least one of the RRBs (ARR11) is able to interact with a number of other TFs with unknown functions belonging to different families (e.g., ATHB-51, ANAC063) [99]. Two other Y2H-based approaches found a set of interacting TFs for ARR2 and ARR14 [100,101]. While these studies have yielded a small but growing list of putative RRB interactors the functional relevance of these interactions is in most cases unclear. However, there are a number of well-documented cases described in the following sections.

## 6. Interaction between RRBs in the Transcriptional Cytokinin Response

**Figure 2:**
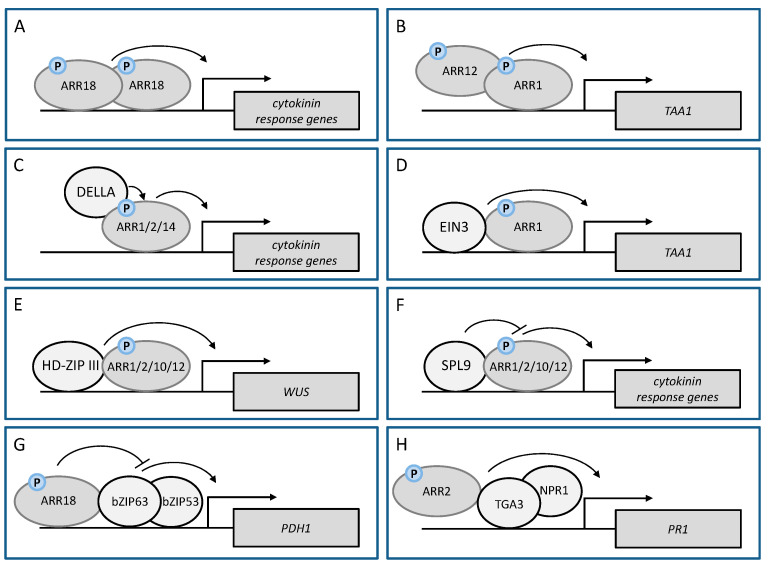
Modulation of transcriptional responses by transcription factor complex formation. (**A**,**B**) Dimerization of RRBs, like the homodimerization of phosphorylated ARR18 and the interaction of ARR12 with ARR1, positively affects their transcriptional activity on cytokinin response genes or specifically *TAA1*. (**C**–**F**) The interaction of other TFs with RRBs either positively or negatively affects the activity of RRBs to regulate the expression of cytokinin response genes (e.g., *TAA1* or *WUS*). (**G**,**H**) RRBs can act as modulators of other TFs and their ability to induce specific response genes. (**G**) Cytokinin-induced phosphorylation of ARR18 leads to its interaction with the bZIP TF-complex bZIP63-bZIP53 dampening their activation of *PDH1* gene expression. (**H**) Phosphorylated ARR2 interacts with TGA3 and positively influences the SA- and NPR1-dependent expression of the pathogen resistance gene *PR1*. Please note that the phosphorylation status of RRBs (P) as shown in the figure has not been analyzed in all cases.

The formation of functional homodimers of an RRB in planta was first shown by FRET–FLIM studies for ARR18 [46]. The homodimerization of ARR18 was required for inducing the expression of the cytokinin target gene *ARR5* in a transient expression system (Figure 2A; Table 1).

Dimerization was dependent on the cytokinin-induced phosphorylation of the conserved aspartate in the receiver domain, which is reminiscent of the phosphorylation-dependent dimerization of bacterial RRs. Interestingly, dimerization of the ARR18 protein was only observed when both monomers were in the same phosphorylation state, and it has been argued that this would increase the precision of the ARR18-mediated transcriptional responses [46]. These data suggested that phosphorylation-dependent dimerization or higher-order oligomerization might represent an essential component of transcriptional regulation of the cytokinin response by RRBs. Another case of functionally relevant RRB homodimers has been reported from rice. EARLY HEADING DATE 1 (EDH1) is a RRB regulating flowering time through the expression of the rice florigen genes *HEADIND DATE 3A* (*HD3A*) and *RICE FLOWERING LOCUS T1* (*RFT1*) [103]. Similar to the case of ARR18, homodimerization of Edh1 was caused by phosphorylation of the aspartate in the receiver domain. Further analysis showed that a 15-amino acid region between the N-terminal receiver domain and the C-terminal DNA-binding domain was crucial for homodimerization and Edh1 activity. Interestingly, Edh1 activity was reduced by an interacting RRA, OsRR1, which retarded flowering. This suggested that cytokinin acts on flowering time through suppression of homodimer formation of the flowering promoting Edh1 [103].

An example for the in planta heterodimerization of RRBs is the formation of an ARR1–ARR12 complex. ARR1 binds specifically to the cytokinin target promoter of *TRYPTOPHAN AMINOTRANSFERASE OF ARABIDOPSIS 1* (*TAA1*), an auxin biosynthesis gene, and induces its expression. The induction was reduced in *arr1* and *arr12* mutants and was almost completely abolished in the *arr1 arr12* double mutant, indicating that both ARR1 and ARR12 are required for *TAA1* expression. Interestingly, ARR12 alone was not able to bind directly to the *TAA1* promoter but had to form a heterodimer together with ARR1 causing enhanced ARR1-dependent expression of *TAA1* (Figure 2B; Table 1). Interaction studies using bimolecular fluorescence complementation (BiFC) showed that the C-terminal parts of these RRBs were sufficient to increase their combined activity [102]. The formation of the ARR1-ARR12 TF complex illustrates how RRB heterodimers can modulate the strength of the cytokinin response and established a link between cytokinin and auxin biosynthesis.

## 7. Interaction of RRBs with Other TFs

Several examples illustrate that interaction of RRBs with TFs of different TF families or with other transcriptional regulators is functionally important. In the cases described below the interacting TF modulates the activity of RRBs in cytokinin signaling or, vice versa, the RRBs modulate the activity of their partner protein in its functional context. The former case is illustrated by the influence of DELLAs and several other TFs on developmental processes regulated by RRBs, while for the latter case, two examples describe that RRBs modulate the activity of bZIPs in the response to different stresses.

### 7.1. DELLA Proteins Act as Co-Regulators of RRBs in the Cytokinin Response

Other hormonal pathways may use RRBs as targets to modulate the cytokinin output. DELLA proteins, negative regulators of the gibberellin (GA) pathway, are transcriptional regulators of the GRAS family without a clearly identified DNA-binding domain. They have been shown to modulate the transcriptional responses of several different signaling pathways [109,110]. A screen for TFs interacting with DELLAs had identified several RRBs (ARR1, ARR2, ARR14) as interactors [104,105]. Thereafter, ChIP analysis in wild type and *RRB* mutant lines revealed that DELLA and RRB proteins share common target genes and that the presence of DELLA proteins at the target sites is dependent on the presence of RRBs. This supported the idea that DELLA proteins are recruited by RRBs to the target promoter of cytokinin-regulated genes [104] (Figure 2C; Table 1). The necessity of the TF complex formation consisting of DELLAs and RRBs for a full cytokinin response was further proven by the analysis of cytokinin-responsive *TCSn:GFP/LUC* reporter gene expression. The reduction of DELLA protein abundance by their GA-dependent degradation reduced the *TCSn:GFP* reporter signal in Arabidopsis root meristems. On the other hand, the co-expression of the DELLA *GIBBERELLIC ACID-INSENSITIVE* (*GAI*) and *ARR1* in tobacco leaves resulted in an enhanced response of the *TCSn:LUC* reporter compared to the response induced by ARR1 alone. This indicated that GAI and RGA enhance the transactivation activity of ARR1 and that ARR1 and DELLA act as transcriptional co-regulators in Arabidopsis requiring their physical interaction and serving as a molecular basis for cytokinin-GA crosstalk. These hormones have often antagonistic effects and in the described scenario GA would lower CK activity by inducing degradation of GAI and RGA [104]. The biological relevance of this mechanism was shown by mutant analysis revealing the necessity of the simultaneous presence of DELLAs and ARR1 to restrict root meristem growth and to promote photomorphogenesis [104].

In an independent study, DELLAs were shown to bind in complex with ARR1 to the promoter of *TAA1* to promote its expression [102]. Yet another TF, ETHYLENE-INSENSITVE3 (EIN3), a positive regulator of the ethylene signaling pathway, can also bind in complex with ARR1 to the *TAA1* promoter (Figure 2C,D; Table 1), in this way enhancing the expression of *TAA1*. Neither DELLAs nor EIN3 were able to induce *TAA1* expression without ARR1. These interactions demonstrate crosstalk between the cytokinin, GA and ethylene pathways to regulate auxin biosynthesis [102].

### 7.2. Dimerization of RRBs with HD-ZIP III and SPL TFs Is Critical for De Novo Shoot Formation

De novo organogenesis is a regenerative process to form new shoots or roots after the loss of a critical portion of the plant body. These newly formed organs can arise directly from parental plants or from callus and need the establishment of meristematic cells [111]. De novo shoot formation is coordinated by cytokinin and depends on the activity of RRBs inducing the expression of WUS [67]. RRBs can bind directly to the WUS promoter and induce its expression to define the stem cell niche. The loss of RRB activity in the SAM of *arr1 arr10 arr12* triple mutants disturbed *WUS* expression and impaired the shoot regeneration capacity [106]. To limit the expression of *WUS* to the center of the regenerating shoot meristem, the activity of RRBs on the *WUS* promoter is restricted by the HD-ZIP III TFs PHABULOSA (PHB), PHAVOLUTA (PHV) and REVOLUTA (REV), which are all targets of miR165/miR166. PHB, PHV and REV are not able to activate the expression of *WUS* alone but physically interact with ARR1 and ARR2 as well as with ARR10 and ARR12. The interactions between these RRBs and PHB or REV were identified by Y2H and confirmed by in vitro pull down, co-immunoprecipitation (CoIP) and bimolecular luminescence complementation (BiLC) [106,107]. The cooperative activity of these RRBs and HD-ZIP III proteins is a critical regulatory step for the activation of *WUS* and the process of shoot regeneration (Figure 2E; Table 1). The reduced abundance of HD-ZIP III TFs by miR165/miR166 overexpression or their loss in the *phb phv rev* triple mutant lowered the expression of *WUS* on cytokinin-containing shoot induction medium (SIM) and consequently reduced the shoot regenerative capacity [106]. It remains to be shown whether their cooperative activity is also required in planta to activate or maintain *WUS* expression during normal development. The observation that the *RRB* and *HD-ZIP III* genes are expressed in a broader domain than *WUS* suggests that additional factors may be involved to limit WUS expression [106].

Physical interaction of RRBs with other TFs is also the reason for the reduced shoot regeneration capacity of old leaves. Here, RRB activity is controlled by the miRNA156/SPL module of the age pathway. The mRNA of several *SQUAMOSA PROMOTER BINDING PROTEIN-LIKE (SPL*) genes encoding TFs are targets of miR156. The age-dependent decline in miR156 abundance leads to a progressive increase of SPL proteins [112,113]. Several SPL TFs (SPL2, SPL9 and SPL10) have the ability to dimerize with RRB proteins (ARR1, ARR2, ARR10, ARR12) thereby dampening their activity on cytokinin target gene promoters (Figure 2F; Table 1). Consequently, RRB activity and the cytokinin response are high in juvenile plants and decrease in older plants which results in a reduction of the regenerative capacity [108]. The RRB transactivation domain was required for interaction with SPLs which occurred independent of the phosphorylation status of the receiver domain, thus differing from the homodimerization of RRBs requiring receiver domain phosphorylation. The RRB-SPL interaction establishes a molecular link between developmental timing and cytokinin-mediated shoot regeneration. Its functional relevance in planta remains to be shown but interference with the role of cytokinin in regulating leaf phase transition and senescence is a possibility.

### 7.3. RRB-bZIP Interaction Plays an Important Role under Water Deficit

Interaction between RRB and a bZIP TF plays also a role in another unrelated stress response. Water deficit due to low water potential or salinity causes in plants the accumulation of the amino acid proline. Proline plays an important role in the osmotic adjustment, increases the membrane stability and is discussed to improve the plant tolerance to water stress [114]. The accumulation of proline is coordinated by the upregulation of proline biosynthesis genes and the downregulation of the *PROLINE DEHYDROGENASE1* (*PDH1*) gene, encoding an enzyme catalyzing the first rate-limiting step of proline breakdown. In the situation of rehydration after dehydration or under conditions of hypoosmolarity, the expression of PDH1 is upregulated, which is largely controlled by bZIP TFs that bind to the ACT-box motif in the *PDH1* promoter [115]. It was shown that a bZIP TF dimer, bZIP53-bZIP63, induces the expression of PDH1 [50]. Cytokinin can negatively influence that regulation via ARR18 depending on the phosphorylation of ARR18. Phosphorylated ARR18 binds to the *PDH1* promoter sequence and in parallel interacts physically with bZIP63 leading to a repression of the bZIP53-bZIP63 complex activity (Figure 2G; Table 1). When the interaction of ARR18 with bZIP63 was disrupted by mutation of the conserved Asp in the receiver domain of ARR18 the repressive effect of ARR18 was negated. In contrast, it was enforced when that Asp residue was replaced by a phosphorylation-mimicking amino acid. The bZIP63-ARR18 interaction specifically affected *PHD1* regulation and did not interfere with the cytokinin gene regulation by ARR18. The ARR18-bZIP interaction is the molecular mechanism through which cytokinin negatively regulates *PDH1* expression to cause a reduced breakdown of proline thus positively affecting seed germination and gene regulation under osmotic stress conditions [50]. It would be interesting to study whether physical interaction between RRBs and bZIP factors is a regulatory mechanism that impacts plant processes more broadly.

### 7.4. ARR2-TGA3 Interaction Affects Plant Defence against the Pathogen Pseudomonas Syringae

The cytokinin level influences Arabidopsis immunity to *Pseudomonas syringae pv. tomato* DC3000 (*Pst* DC3000) [19]. An increased cytokinin content resulted in an enhanced resistance of Arabidopsis against *Pst* DC3000 while a reduced cytokinin content or signaling led to an increased susceptibility. Pathogen infection causes an increase in the concentration of salicylic acid (SA). SA mobilizes the transcriptional coactivator NONEXPRESSOR OF PR GENES (NPR1), which recruits the SA-responsive TF TGA1A-RELATED GENE 3 (TGA3) of the bZIP family to bind to the promoter of the *PATHOGENESIS-RELATED 1* (*PR1*) gene, a marker gene for systemic acquired resistance (SAR), and induces its expression [116]. This process can be modulated by ARR2, which forms a complex together with TGA3 as shown by CoIP and BiFC. This complex is recruited to the *PR1* promoter (Figure 2H; Table 1), where it binds via TGA3 to the TGA-binding *cis*-element LS7, thus enhancing the expression of *PR1* and thereby providing resistance against *Pst* DC3000. Cytokinin-dependent phosphorylation of ARR2 but also higher SA signaling enhanced the binding of the ARR2-TGA3 complex to the *PR1* promoter [19]. In contrast, ARR2 was not able to bind to the *PR1* promoter on its own. Importantly, the binding activity of ARR2 to its cognate cytokinin target promoters was not affected in NPR1 or TGA3 mutants [19]. Taken together, these results show that cytokinin modulates SA-dependent defense signaling to augment resistance against *Pst* DC3000, a process which is mediated by the interaction between TGA3 and ARR2.

## 8. Conclusions

Numerous reports show that TFs can interact with other TFs and that this mode of action can regulate or modify their transcriptional activity. RRBs, the cytokinin output TFs, are likewise able to interact specifically with other TFs belonging to different families, including the AP2/ERF family members CRFs, the bZIPs TGA3 and bZIP63, DELLAs and SPLs to regulate and specify the transcriptional output of cytokinin signaling (Table 1). These interactions lead to the formation of RRB-TF-complexes which either positively or negatively influence the transcriptional activity of the RRBs or change the transcriptional properties of the interacting TF (Figure 2). In this way, the formation of RRB-TF-complexes is a regulatory mechanism to make cytokinin signaling accessible for the influence of other signaling pathways and/or to modify the output of other signaling pathways. This mode of action certainly contributes to enable different RRBs with partially overlapping expression patterns and functionalities to generate complex and specific transcriptional outputs.

Different structural components are implicated in the RRB-TF interaction. Homo- and heterodimerization depend on the C-terminal part containing the DNA-binding domain and the transactivation domain. In these cases, interaction takes place only in the phosphorylated state. In distinction, the RRB transactivation domain was also required to interact with DELLAs and SPLs [104,108] but the interaction took place independent of the phosphorylation status of the receiver domain. In case of Edh1 dimerization a short stretch of 15 aa between the receiver domain and the DNA-binding domain was essential for dimer formation. Resolving the three-dimensional structure of RRBs would be helpful to model the interaction as has been carried out for receptor and phosphotransmitter interaction [117].

Despite increasing knowledge about the interaction of RRBs with other TFs only relatively few well-documented examples illustrate their potential to regulate cytokinin activities. It is therefore of great interest to gain more knowledge about RRB-TF interaction. Surprisingly few candidate interaction partners have been identified in large scale protein–protein interaction screens [99,100,101,118]. This is partly due to the fact that only few (or no) RRBs were included as baits in several of these screens. Furthermore, this type of interaction screens may yield variable results. For example, Y2H analysis in search for TFs interacting with DELLA proteins yielded 57 TF interaction partners of GAI [105]. A similar number of 71 interaction partners of GAI was found in the CrY2H-Seq-based approach [99] but only one interaction (GAI-PIF3) was found in both analyses using distinct but overlapping TF libraries. In addition, known interaction partners of RRBs were not identified in these large-scale interaction screens. This shows the limitations of such explorative large-scale analyses and argues for a more targeted approach to deepen our understanding of cytokinin activities downstream of the two-component signaling system. Indeed, screening protein interaction between several Arabidopsis RRBs and a TF library [119] has identified numerous putative interaction partners (J.E.L., Mhyedeen Halawa and T.S., unpublished result) and will be a rich source for further investigation.

A further aspect deserving attention in addition to physical interaction of TFs is the interaction of TF pairs that bind cooperatively to DNA. A systematic analysis in human cells has revealed several hundred TF–TF interactions, indicating cooperativity in DNA-binding between TFs of diverse families, most of them were previously unknown [120]. These interactions did mainly not rely on protein–protein interaction but were predominantly mediated by DNA. Notably, most TF pair sites recognized composite DNA binding sites that were markedly different from the individual TF binding motifs. This could at least partly explain the lack of identifiable CRMs in numerous cytokinin response genes.

## Figures and Tables

**Table 1:** Interaction of RRBs with other transcriptional regulators controls diverse processes.

RRB	Interaction Partner	Activity	Functional Context	References
**Formation of RRB homo- and heterodimers**				
ARR18	ARR18	ARR18 homodimerization positively regulates the cytokinin response	cytokinin response	[46]
ARR1	ARR12	ARR1-ARR12 heterodimerisation positively affects transcriptional activation of *TAA1* by cytokinin	auxin biosynthesis	[102]
EDH1	EDH1	EDH1 homodimerization activates rice florigen genes	regulation of flowering	[103]
**RRBs interact with other transcriptional regulators to regulate cytokinin target genes**				
ARR1 ARR2 ARR14	GAI and RGA (DELLA)	RRB/DELLA complex positively regulates the cytokinin response	root meristem, photomorphogenesis	[102,104,105]
ARR1	EIN3	ARR1/EIN3 complex positively affects the transcriptional activation of *TAA1* by cytokinin	auxin biosynthesis	[102]
ARR1 ARR2 ARR10 ARR12	PHB, PHV, REV (HD-ZIP III)	RRB/HD-ZIP III complex activates WUS expression	de novo SAM formation	[106,107]
ARR1 ARR2 ARR10 ARR12	SPL9	RRB/SPL9 complex negatively regulates the cytokinin response	age-dependent decline in de novo SAM formation	[108]
**RRBs interact with other transcriptional regulators to regulate target genes of other signaling pathways**				
ARR2	TGA3	ARR2 promotes the activity of TGA3/NPR1 on the *PR1* promoter	pathogen resistance against *Pst* DC3000	[19]
ARR18	bZIP63	ARR18 dampens the activity of the bZIP63/bZIP53 complex on the *PDH1* promoter	plant stress responses	[50]
